# Oral Microbiota Alterations in Subjects with SARS-CoV-2 Displaying Prevalence of the Opportunistic Fungal Pathogen *Candida albicans*

**DOI:** 10.3390/microorganisms12071356

**Published:** 2024-07-02

**Authors:** Areej A. Alfaifi, Johanna B. Holm, Tristan W. Wang, Jonathan Lim, Timothy F. Meiller, Peter Rock, Ahmed S. Sultan, Mary Ann Jabra-Rizk

**Affiliations:** 1 Department of Oncology and Diagnostic Sciences, School of Dentistry, University of Maryland Baltimore, Baltimore, MD 21201, USA; aalfaifi@umaryland.edu (A.A.A.); twang2@umaryland.edu (T.W.W.); tfmeiller@umaryland.edu (T.F.M.); asultan@umaryland.edu (A.S.S.); 2 Department of Restorative and Prosthetic Dental Sciences, College of Dentistry, King Saud bin Abdulaziz University for Health Sciences, Riyadh 11426, Saudi Arabia; 3 King Abdullah International Medical Research Center (KAIMRC), Riyadh 11481, Saudi Arabia; 4 Institute for Genome Sciences, School of Medicine, University of Maryland, Baltimore, MD 21201, USA; jholm@som.umaryland.edu (J.B.H.); jolim@som.umaryland.edu (J.L.); 5 Department of Microbiology and Immunology, School of Medicine, University of Maryland, Baltimore, MD 21201, USA; 6 Greenebaum Cancer Center, University of Maryland, Baltimore, MD 21201, USA; 7 Department of Anesthesia, School of Medicine, University of Maryland, Baltimore, MD 21201, USA; prock@som.umaryland.edu

**Keywords:** oral, microbiome, COVID-19, SARS-CoV-2, *Candida albicans*

## Abstract

The oral cavity remains an underappreciated site for SARS-CoV-2 infection despite the myriad of oral conditions in COVID-19 patients. Recently, SARS-CoV-2 was shown to replicate in the salivary gland cells causing tissue inflammation. Given the established association between inflammation and microbiome disruption, we comparatively profiled oral microbial differences at a metagenomic level in a cohort of hospitalized COVID-19 patients and matched healthy controls. Specifically, we aimed to evaluate colonization by the opportunistic fungal pathogen *Candida albicans*, the etiologic agent of oral candidiasis. Comprehensive shotgun metagenomic analysis indicated that, overall, COVID-19 patients exhibited significantly reduced bacterial and viral diversity/richness; we identified 12 differentially abundant bacterial species to be negatively associated with COVID-19, and the functional pathways of certain bacteria to be highly associated with COVID-19 status. Strikingly, *C. albicans* was recovered from approximately half of the COVID-19 subjects but not from any of the healthy controls. The prevalence of *Candida* is likely linked to immune hypo-dysregulation caused by COVID-19 favoring *Candida* proliferation, warranting investigations into the interplay between *Candida* and SARS-CoV2 and potential therapeutic approaches directed toward oral candidiasis. Collectively, our findings prompt a reassessment of oral opportunistic infection risks during COVID-19 disease and their potential long-term impacts on oral health.

## 1. Introduction

The oral cavity remains an underappreciated site for SARS-CoV-2 infection despite the myriad of oral conditions observed in COVID-19 patients. Oral manifestations observed in documented cases of COVID-19 include non-specific white and erythematous plaques, gustatory dysfunction, aphthous-like ulcerations, bullae, necrotizing gingivitis, coinfections due to hypergrowth of opportunistic oral pathogens, and salivary gland alterations [1,2,3]. The salivary glands were identified to be a potential target for SARS-CoV-2 infection as several recent studies demonstrated the concomitant expression of ACE2/transmembrane serine proteases 2 (TMPRSS2) in the epithelial cells of the oral mucosa and the of salivary glands [3,4]. These studies strongly imply that the oral cavity might be more susceptible to viral infiltration.

The healthy microbiota in the oral environment are dynamic and complex microbial communities composed of bacteria, fungi, and viruses tightly coexisting with each other [5]. The interactions of the host with the microbiota are complex, numerous, and bidirectional because the microbiota affects numerous biological functions important for maintaining health and immune homeostasis [6,7]. In fact, commensal microbiota are thought to be the main drivers in shaping protective immune responses at barrier tissues [5,8]. However, the diverse microbial groups can have both synergistic and antagonistic interactions where suppression of one group can affect the growth of another [9]. Dysbiosis, or the alteration of microbiota components can contribute to the initiation or advancement of various diseases [10]; therefore, considering the crucial role of microbiota in regulating and modulating the immune system and inflammatory processes, comparing the oral microbiomes of COVID-19 patients with healthy controls would improve our understanding of the impacts of COVID-19 infections on dysbiosis.

Several recent studies have reported on the potential association between COVID-19 and the onset of microbiome dysbiosis [10,11,12]. A study by Shengli et al. (2021) [13] described distinct oropharyngeal microbiota in COVID-19 patients, characterized by enrichment of opportunistic pathogens such as *Megasphaera* and *Veillonella*, and depletion of *Streptococcus*, *Rothia*, and *Pseudopropionibacterium.* In another study, next-generation metagenomic sequencing demonstrated the presence of *Veillonella*, *Capnocytophaga*, and other oral opportunists in the bronchoalveolar lavage fluid of COVID-19 patients [14]. Importantly, metagenomic analyses of those infected with SARS-CoV-2 frequently show abnormally high reads of cariogenic and periodontopathic bacteria with an increased abundance of *Prevotella intermedia*, *Streptococci*, *Lactobacillus*, *Porphyromonas*, *Capnocytophaga*, *Aggregatibacter*, *Abiotrophia*, and *Atopobium* suggesting an association between these bacteria and SARS-CoV-2 infection [15]. In addition, Shengli et al. (2021) [13] reported that in COVID-19 patients, the oropharynx shows notable enrichment of various members of the *Veillonella* genus, particularly *Veillonella parvula*. Interestingly, the *Veillonella* genus was also found to be in elevated proportions in the bronchoalveolar lavage fluid (BALF) of individuals affected by COVID-19 [14]. Combined, these findings suggest that the oral cavity acts as a natural source for pathogens, potentially leading to co-infections within the lungs of individuals affected by COVID-19. However, despite the intense interest in characterizing the bacterial microbiota during COVID-19 disease, the mycobiome (fungal) and virome (viral) components of the oral microbiome have not garnered enough attention, although some reports have indicated increases in fungal species in patients with COVID-19 [16,17]. Among the fungi, *Candida albicans* (*C. albicans*) is the most frequently isolated fungal species. Although a common member of the human microbiota for at least half of the human population, this opportunistic species is highly adaptive with the ability to rapidly transition from commensal to pathogen [9,18]. Oral candidiasis (thrush), a mucosal infection primarily affecting the tongue dorsum, is the result of overgrowth of *C. albicans*. In fact, oral candidiasis is the most prevalent oral condition, particularly among immunocompromised patients, and shifts in the local microbial communities are thought to be the trigger of disease pathology [18,19,20].

In light of the recent demonstration of the presence of proliferating SARS-CoV-2 in salivary gland epithelial cells—resulting in inflammation likely linked to immune dysregulation [3]—in this study, we aimed to characterize the oral microbiota in subjects infected with SARS-CoV-2, focusing on assessing the prevalence of *Candida* and potential predisposition to oral candidiasis. Given the role of the microbiome in mediating inflammation and vice versa, studies characterizing shifts in the oral microbiota within the context of the presence of SARS-CoV-2 are clearly warranted. To that end, we performed shotgun metagenomic sequencing on oral samples recovered from a cohort of hospitalized COVID-19 patients. The comprehensive analysis included a comparison of microbial diversity, relative abundances of bacteria, viruses, and fungi, and metagenomic functions between the oral microbiomes of COVID-19 patients and matched healthy subjects. Importantly, all study subjects were also sampled and cultured for assessment of *Candida* colonization as a marker for susceptibility for the development of oral candidiasis.

## 2. Materials and Methods

### 2.1. Subjects and Clinical Samples

A total of 26 adult COVID-19 patients (29 samples; 2 samples were recovered for 3 of the patients) hospitalized at the University of Maryland Medical Center and a control group of 21 healthy volunteers attending the University of Maryland School of Dentistry were included in this study (subject demographics and characteristics are presented in Table 1). The University of Maryland Baltimore Institutional Review Board approved this study, and informed consent was obtained from all subjects. Oral swab samples were collected from hospitalized COVID-19 patients upon admission following the diagnosis of SARS-CoV-2 infection. Depending on sample availability, serial samples were collected from some patients during their hospitalization period. Oral mucosal surfaces, including the internal surfaces of both cheeks, above and below the tongue, and the hard palate were swabbed for 20 s. The oral swab specimens were collected using Norgen Biotek’s DNA preservative system (Norgen Biotek Corp., Thorold, ON, Canada) and stored at room temperature until analysis. Inclusion criteria for COVID-19 patients was a positive test indicating the presence of the virus in the nares and oropharyngeal cavity; exclusion criteria for control subjects included recent COVID-19 diagnosis and any reported oral pathological conditions such as oral candidiasis or recent antifungal therapy.

### 2.2. Evaluation of Oral Fungal Colonization

In order to comparatively assess *Candida* colonization status, oral swabs from all sampled subjects were immediately cultured on fungal Yeast Peptone Dextrose (YPD) agar media (Difco Laboratories, Detroit, MI, USA) and plates were incubated at 35 °C for 24–48 h. All fungal colonies were speciated using the chromogenic media CHROMagar Candida (DRG International, Springfield NJ, USA) and species identified based on colony color.

### 2.3. DNA Extraction

Genomic DNA was extracted using the Quick-DNA Fungal/Bacterial Microprep Kit (Zymo ResearchCorp., Irvine, CA, USA) according to manufacturer recommendation. Both positive and negative controls (Zymo ResearchCorp.) were included in the DNA extraction process and DNA concentration in the samples was determined using the Bioanalyzer 2100 DNA 1000 chip (Agilent, Santa Clara, CA, USA).

### 2.4. Shotgun Metagenomics Sequencing

Shotgun metagenomic sequence libraries were constructed from the DNA extracts using Illumina Nextera XT Flex kits (San Diego, CA, USA) according to the manufacturer’s recommendations and then sequenced on an Illumina HiSeq 4000 platform (150 bp paired-end mode) at the Genomic Resource Center at the University of Maryland School of Medicine. Each sample was uniquely barcoded in each HiSeq 4000 lane, yielding an average of 40 million read pairs for each sample. The sequencing data are publicly available (https://www.ncbi.nlm.nih.gov/sra/PRJNA997379, (accessed on 1 May 2024)).

### 2.5. Bioinformatics and Statistical Analysis

Quality control of each metagenome was performed using BBMap (v38.87) [21] and bioBakery3 suite2 [22]. Identical duplicates were removed using the Clumpify tool in “dedupe” mode allowing 0 substitutions. BBDuK was run to remove reads from the phiX spike-in and synthetic molecules (k = 31). Trimming of low-quality bases and removal of adapters, short reads, and filtering of human reads was performed using Kneaddata (v0.10.0).

Taxonomic and functional profiling was performed using tools from bioBakery 3 [22]. Taxonomic profiles were estimated by mapping reads to clade-specific marker genes using Metaphlan (version 4.0.6) with database version 3.1. Gene families and pathways were profiled using HUMAnN (version 3.7), ChocoPhlAn nucleotide database (version 3.1), and the Uniref90 protein database [23]. The reads and taxonomic profiles were provided as input to HUMAnN. Pathway abundances and coverages were computed based on MetaCyc pathway definitions. Gene family and pathway abundances were depth-normalized to CPM (counts per million) and relative abundances using the “humann_renorm_table” script included with HUMAnN.

Statistical analyses were performed using R (version 3.6.0). Comparisons of α-diversity metrices were performed using mixed-effect logistic regression where the outcome was the Shannon diversity (or mean Shannon diversity in the case of repeated measures), the predictor was COVID-19 status, *Candida* infection, or COVID-19 and *Candida* co-infection, and a random effect accounting for age, race, and gender via a matched value was included. β-diversity was evaluated using Bray–Curtis distances (vegan package) of sample taxonomic compositions and hierarchical clustering via Ward linkage (function hclust). Resulting clusters were tested for associations with COVID-19 status using mixed-effect logistic regression where the outcome was the microbiome cluster (Cluster 3 or not), the predictor was COVID-19 status (ref: uninfected), and a random effect was incorporated to account for sample matching.

To identify associations between specific taxa, functional gene pathways of specific taxa, and COVID-19 status, the metaphlan3 abundance data (taxa-specific) and the RPK count data of gene pathways with corresponding taxonomic information were evaluated for associations with COVID-19 status in separate models. For taxon-specific analysis, taxa observed in at least 10% of samples (n = 5) were tested for associations. For the pathway analysis, KEGG orthologs with taxonomic annotations present in at least 40% of samples were evaluated. For each model, abundance data were normalized for differences in coverage using the “poscounts” estimator in the R package DESeq2 (v1.44.0). This approach addresses pathways with zeros by calculating a modified geometric mean as the n-th root of the product of the non-zero counts. Mixed-effect linear regression [24] was used to model the relationship between functional pathways and COVID-19 status and associations were tested for significance using the Bayes test [25]. Models accounted for the match variable, *Candida* infection, and repeated measures (the “block factor” was the host subject ID). All *p*-values were adjusted for multiple testing with BH correction with a false discovery rate threshold of 5%, and log2 differences in taxon-specific ortholog abundances are reported.

## 3. Results

### 3.1. Significant Prevalence of Oral Candida Colonization in COVID-19 Patients

Of the 26 COVID-19 subjects included in the study, 22 samples were cultured for fungal growth (no samples were acquired from 8 subjects due to patient medical status); of the 22 samples, 10 (45%) were positive for *Candida* with growth ranging from moderate to high levels with *C. albicans* as the only species identified. In contrast, no *Candida* was recovered from any of the 21 samples cultured from healthy control subjects.

### 3.2. Significant Differences in Diversity of the Oral Microbiome of COVID-19 Patients Compared to Healthy Subjects

**Figure 1:**
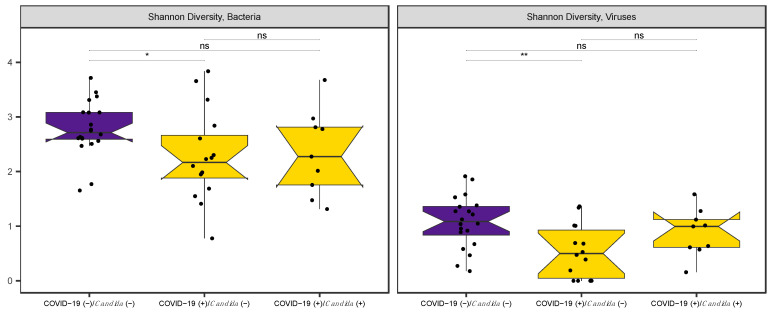
Oral bacterial and viral alpha-diversity is lower in COVID-19-positive patients without *Candida* infection relative to healthy, COVID-19-negative controls. * *p* < 0.05; ** *p* < 0.01; ns not significant.

There was less viral α-diversity than bacterial diversity among all patients (Figure 1). On average, bacterial and viral α-diversity were 16% and 17% lower in COVID-19-positive patients compared to COVID-19-negative patients, respectively (p_bacterial_ = 0.02 and p_viral_ < 0.01). These differences were significant even after adjusting for subject-matched variables. *Candida* infection was not associated with α-diversity. When further stratified by *Candida* infections, the oral microbiota of those with COVID-19 and *Candida* co-infection were not significantly less diverse than COVID-19-negative patients (Figure 1), likely reflecting the few patients with COVID-19 and *Candida* co-infections (n = 9).

**Figure 2:**
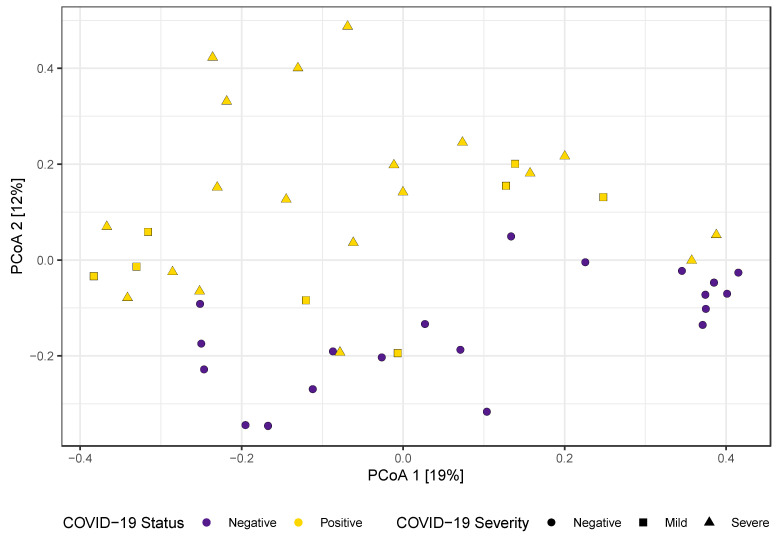
PCoA of Bray–Curtis dissimilarities distinguish the oral microbiomes by COVID-19 status.

β-diversity was also associated with COVID-19 status (Figure 2). Principle coordinates analysis of Bray–Curtis dissimilarities captured 31% of the variance in the first two axes. Specifically, PCoA2 was significantly associated with COVID-19 status (F = 25, *p* < 0.001). Among COVID-19-positive individuals, the severity of the infection was not associated with α- or β-diversity.

**Figure 3:**
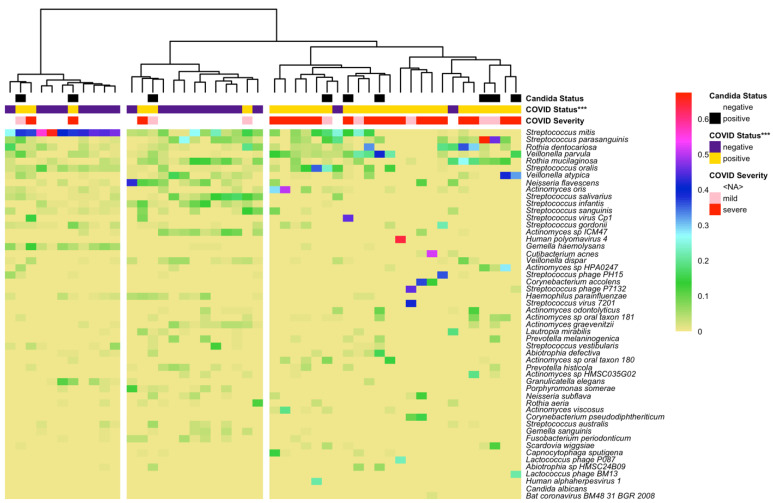
Oral microbiome composition was significantly associated with COVID-19 status (***: *p* < 0.001). Shown are the fifty most abundant taxa, *Candida albicans*, and *Bat coronavirus*, and their proportions in each sample.

The most abundant taxa included bacteria, viruses, and phages (Figure 3). Hierarchical clustering of β-diversity resulted in three major clusters: one predominated by *Streptococcus mitis*, another with high diversity but no predominance of *S. mitis*, and the third with relatively fewer species and no predominance of *S. mitis*. This cluster was significantly associated with COVID-19 status after adjusting for the match (*p* < 0.001). Many samples in this cluster had a predominant species though the species was not the same. This was reflected in the taxon-specific analysis—of 182 taxa tested, only *S. mitis* was significantly associated with COVID-19 status, specifically, it was more abundant among COVID-19-negative participants (Table S1). *Candida* infection was not associated with β-diversity. Notably, some bacterial species were observed in most samples regardless of COVID-19 status, including *Streptococus parasanguinis*, *Rothia dentocariosa*, and *Veillonella parvula*. In terms of fungal species, we found increased abundances of the fungal genera *Malassezia*, *Candida,* and *Saccharomyces* in samples from COVID-19 patients.

### 3.3. Changes in Bacterial Functions Associated with COVID-19

**Figure 4:**
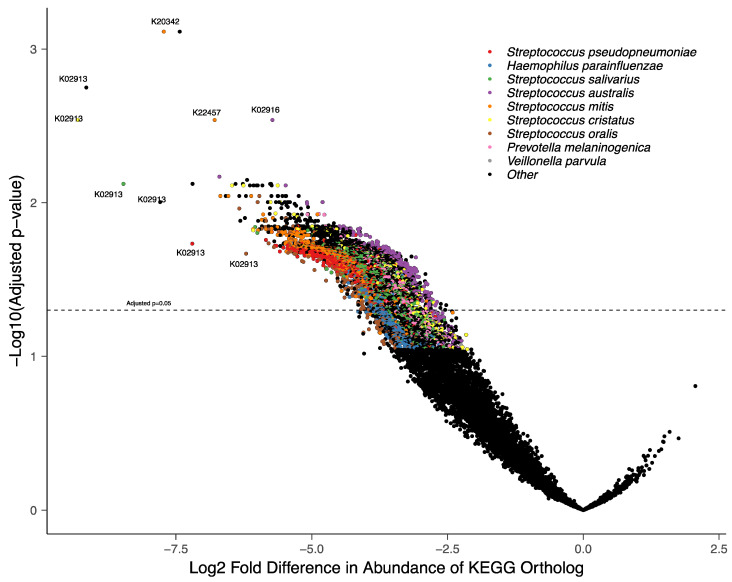
Functional pathways of certain oral bacteria were highly associated with COVID-19 status. Shown are the 23 most associated pathways with taxonomic annotation.

To gain insight into functional changes within the COVID-19-associated oral microbiome, we studied the Kyoto Encyclopedia of Genes and Genomes (KEGG) gene orthologs enriched in the oral microbiota of the COVID-19 patients compared to the controls (Figure 4). Of 11,367 orthologs tested, 3350 were significantly associated with COVID-19-negative individuals and were from multiple *Streptococcus* species, including *S. mitis*, *Haemophilus parainfluenzae*, and *Prevotella melaninogenica*. Of note, K02913—the large subunit of the ribosomal protein—from multiple species was associated with COVID-19-negative controls reflecting the lower diversity of COVID-19-positive oral microbiota. Furthermore, an ortholog of nicotinamide mononucleotide transporter (*pnuC*, K03811) from *P. melaninogenica* was more abundant in COVID-19-negative controls, suggesting this species may be able to uptake exogenous nicotinamide riboside, a precursor of NAD+ (Table S2).

## 4. Discussion

SARS-CoV-2 may interact with the oral microbiota via mechanisms involving changes in cytokines, T cell responses, and oral microbiome changes. In fact, there have been a large number of COVID-19 patients coinfected with other viruses, fungi, and bacteria—fungal coinfection specifically is a suggested etiology for COVID-19-related oral manifestations [26,27,28]. While the respiratory and gastrointestinal tracts’ microbiome within the context of COVID-19 disease have been intensely studied, little is known about the impact on the health of the oral cavity despite the numerous oral manifestations observed in COVID-19 patients. Our study aimed to comprehensively characterize the overall microbiome, including viruses and fungi, with a focus on assessing colonization by the opportunistic pathogen *C. albicans* and the risk for the development of oral candidiasis.

Collectively, our findings indicate that SARS-CoV-2 infection alters the composition of the overall oral microbiota. Specifically, we demonstrated significant differences in the diversity and richness of microbial communities in COVID-19 patients compared to matched healthy controls, potentially indicating shifts in the oral microbiota. Only *Streptococcus mitis* demonstrated significantly lower abundances in COVID-19 patients compared to healthy controls but the abundances of species belonging to the genera *Gemella*, *Streptococcus*, *Haemophilus*, *Lautropia*, *Mogibacterium*, *Fusobacterium*, *Actinomyces*, *Leptotrichia*, *Porphyromonas*, *Rothia*, *Neisseria*, *Oribacterium*, and *Corynebacterium* trended towards being lower among COVID-19 patients, consistent with some reports from previous studies [13,29,30]. On the other hand, at the species level, *Veillonella parvula* and *Actinomyces* sp. oral taxon 181, and *Anaeroglobus geminatus* trended towards higher abundances in COVID-19 patients, though this was not statistically significant. Notably, *Anaeroglobus geminatus* is of particular significance as it has been described to be a potential contributor to the microbial shift associated with periodontitis, a common oral inflammatory disease [31]. Although our analysis revealed a higher abundance of *Veillonella parvula* regardless of COVID-19 status, some studies have reported an overrepresentation of *Veillonella* in the oral microbiota of COVID-19 patients [13,32]. In fact, *Veillonella parvula* was found to be highly enriched in the oropharynx of COVID-19 patients [13], confirming that the oral cavity acts as a natural reservoir for pathogens, possibly leading to the development of co-infections in the lungs of individuals with COVID-19. Similar to what was observed with bacterial diversity, relative abundance profiles of the viral component also indicated a significant decrease in viral α-diversity among COVID-19 patients. Although analysis indicated no significant associations between any viruses and COVID-19 status, human betaherpes virus trended towards higher in COVID-19 patients.

In comparing the oral mycobiome in COVID-19 patients and healthy control subjects, our findings revealed increased abundances of the fungal genera *Malassezia*, *Candida*, and *Saccharomyces* in samples from COVID-19 patients. An increase in fungal species such as *Candida*, *Saccharomyces*, and *Simplicillium* in individuals with COVID-19 infection has been reported [10,33]; although *Saccharomyces* is not considered a pathogen, this fungus possesses varying biological effects in health and disease and has been suggested to impact host purine metabolism and intestinal barrier function [34]. Perhaps most revealing from our study is the results of fungal culturing of obtained oral samples from the COVID-19 and control cohorts, which demonstrated approximately 45% of COVID-19 subjects to be colonized by *C. albicans*, some at a very high level. Although *Candida* is a commensal oral colonizer, it is an opportunistic pathogen able to rapidly transition from commensal to pathogen under conditions of immune disruption, or changes in the host environment causing mucosal infections or life-threatening invasive candidiasis, particularly in immunocompromised patients [18]. Notably, however, although a member of the normal oral microbiota in some healthy individuals, *Candida* was not recovered from any of the healthy subjects. As immunosuppression is often associated with SARS-CoV-2 infection, a putative pathogenetic mechanism for *Candida* co-infections in SARS-CoV-2 positive patients could be linked to immune hypo-dysregulation and inflammatory hypo-reactions caused by COVID-19 favoring *Candida* proliferation and infection, similar to what happens in HIV-positive subjects [35]. The stark prevalence of this fungal species is of important clinical relevance as they may indicate that individuals infected with SARS-CoV-2 could be at risk for the development of oral candidiasis, potentially warranting prophylactic therapeutic antifungal intervention. Significantly, even post-COVID-19 recovery, it is suggested that subjects may remain predisposed to oral candidiasis as part of the long COVID syndrome [36] (https://www.covid.gov/be-informed/longcovid/about#term, (accessed on 14 February 2024)). Therefore, in-depth investigations focusing on the interplay between oral candidiasis and SARS-CoV2 and therapeutic approaches directed toward oral candidiasis in COVID-19 are warranted. We would like to mention that despite the demonstrated prevalence of *C. albicans* in samples from COVID-19 patients upon culturing, although *Candida* was detected in sequencing, the coverage was very low because the number of host and bacterial cells far surpasses the number of *Candida* cells.

Given the disparate compositions of COVID-19-positive samples, it is unsurprising that few pathways were associated with COVID-positive status. However, it is notable that among the top pathways found to be associated with COVID-19-negative status was an ortholog of the nicotinamide mononucleotide transporter *pnuC* from *P. melaninogenica*, a conditionally pathogenic anaerobic bacteria, which is mainly responsible for oral infections and inflammation [37]. *PnuC* is a membrane protein involved in the nicotinamide adenine dinucleotide (NAD+) salvage pathway; NAD is a necessary cofactor present in all living cells and some bacteria use the salvage pathway to import nicotinamide riboside via the membrane importer [38,39]. Homologs of this importer have been identified in several bacterial species and a functional analysis of PnuC in *Streptococcus suis* demonstrated that this transporter is important for oxidative stress tolerance [38]. Although the significance is not clear, these findings suggest that *P. melaninogenica* may be able to uptake exogenous nicotinamide riboside, a precursor of NAD+.

Collectively, the findings from this study demonstrate significantly reduced bacterial and viral diversity/richness in the microbiota of COVID-19 patients. Importantly, we demonstrate significant prevalence in *C. albicans*, which may lead to a reassessment of risks for the susceptibility of the development of oral opportunistic infections and inflammatory conditions during the course of COVID-19 disease.

## Figures and Tables

**Table 1:** Characteristics of hospitalized COVID-19 patients and matched healthy controls.

	COVID-19 Patients	Healthy Controls
**Subjects (n)**	26	21
**Samples (n)**	29	21
**Sex**		
Male	15	10
Female	11	11
**Race**		
Black	9	7
White	17	14
**Age range (yrs)**	29–76	27–71
**Symptomatic**	17	-
ICU	6	-
O₂ supply	12	-
**Asymptomatic**	9	-

## Data Availability

The sequencing data are publicly available (https://www.ncbi.nlm.nih.gov/sra/PRJNA997379, accessed on 1 May 2024).

## Supplementary Materials

The following supporting information can be downloaded at: https://www.mdpi.com/article/10.3390/microorganisms12071356/s1, Table S1: Taxa associated with COVID-19 status; Table S2: Functional pathways and their species associated with COVID-19 status
